# Calcium Handling in Human Induced Pluripotent Stem Cell Derived Cardiomyocytes

**DOI:** 10.1371/journal.pone.0018037

**Published:** 2011-04-01

**Authors:** Ilanit Itzhaki, Sophia Rapoport, Irit Huber, Itzhak Mizrahi, Limor Zwi-Dantsis, Gil Arbel, Jackie Schiller, Lior Gepstein

**Affiliations:** 1 Sohnis Family Research Laboratory for Cardiac Electrophysiology and Regenerative Medicine, Bruce Rappaport Faculty of Medicine, Technion - Israel Institute of Technology, Haifa, Israel; 2 Department of Biophysics Physiology, Technion - Israel Institute of Technology, Haifa, Israel; University of Southern California, United States of America

## Abstract

**Background:**

The ability to establish human induced pluripotent stem cells (hiPSCs) by reprogramming of adult fibroblasts and to coax their differentiation into cardiomyocytes opens unique opportunities for cardiovascular regenerative and personalized medicine. In the current study, we investigated the Ca^2+^-handling properties of hiPSCs derived-cardiomyocytes (hiPSC-CMs).

**Methodology/Principal Findings:**

RT-PCR and immunocytochemistry experiments identified the expression of key Ca^2+^-handling proteins. Detailed laser confocal Ca^2+^ imaging demonstrated spontaneous whole-cell [Ca^2+^]_i_ transients. These transients required Ca^2+^ influx via L-type Ca^2+^ channels, as demonstrated by their elimination in the absence of extracellular Ca^2+^ or by administration of the L-type Ca^2+^ channel blocker nifedipine. The presence of a functional ryanodine receptor (RyR)-mediated sarcoplasmic reticulum (SR) Ca^2+^ store, contributing to [Ca^2+^]_i_ transients, was established by application of caffeine (triggering a rapid increase in cytosolic Ca^2+^) and ryanodine (decreasing [Ca^2+^]_i_). Similarly, the importance of Ca^2+^ reuptake into the SR via the SR Ca^2+^ ATPase (SERCA) pump was demonstrated by the inhibiting effect of its blocker (thapsigargin), which led to [Ca^2+^]_i_ transients elimination. Finally, the presence of an IP3-releasable Ca^2+^ pool in hiPSC-CMs and its contribution to whole-cell [Ca^2+^]_i_ transients was demonstrated by the inhibitory effects induced by the IP3-receptor blocker 2-Aminoethoxydiphenyl borate (2-APB) and the phosopholipase C inhibitor U73122.

**Conclusions/Significance:**

Our study establishes the presence of a functional, SERCA-sequestering, RyR-mediated SR Ca^2+^ store in hiPSC-CMs. Furthermore, it demonstrates the dependency of whole-cell [Ca^2+^]_i_ transients in hiPSC-CMs on both sarcolemmal Ca^2+^ entry via L-type Ca^2+^ channels and intracellular store Ca^2+^ release.

## Introduction

The breakthrough technology introduced by Takahashi and Yamanka in 2006 enables the derivation of pluripotent stem cells by reprogramming of somatic cells with a set of transcription factors [1]. Application of this reprogramming strategy to human fibroblasts resulted in the establishment of human induced pluripotent stem cells (hiPSCs) [2], [3]. The hiPSC lines generated were demonstrated to closely-resemble the previously described human embryonic stem cell (hESC) lines [4], including in their ability to differentiate into advanced cell-derivatives of all three germ layers.

Only a limited number of studies described the ability to direct hiPSC differentiation towards the desired cardiac-lineage [5], [6], [7], [8]. As a consequence very little is known about these human iPSC-derived cardiomyocytes' (hiPSC-CMs) functional capabilities, and even less is known about their excitation-contraction (E-C) coupling and Ca^2+^-handling properties [5]. Thorough characterization of the functional nature of hiPSC-CMs must be conducted before these cells can be considered as candidates for the emerging fields of regenerative medicine (potentially providing autologous cardiomyocytes for myocardial regeneration strategies) and personalized medicine (for the derivation of patient/disease-specific *in-vitro* models of human cardiac tissue). The suitability of hiPSC-CMs for such tasks depends, in part, on their contractile characteristics which in turn greatly depend on the Ca^2+^-handling nature of these cells.

In adult ventricular cardiomyocytes, Ca^2+^-handling displays a well-defined sequence of events. Ca^2+^ influx into the cells via depolarization-activated L-type Ca^2+^ channels serves as an initial trigger that is then amplified several folds by sarcoplasmic reticulum (SR) Ca^2+^-store release via Ca^2+^-sensitive ryanodine receptors (RyRs); a process known as Ca^2+^ induced Ca^2+^ release (CICR) [9], [10]. Nevertheless, exceptions to the CICR model have been reported in different species and in developing cardiomyocytes with whole-cell [Ca^2+^]_i_ transients being derived solely from Ca^2+^ influx through the membrane Ca^2+^ channels [11], [12], [13] or by spontaneous release from the intracellular Ca^2+^ stores [14].

In the current study, we tested the hypothesis that whole-cell [Ca^2+^]_i_ transients in hiPSC-CMs are dependent on both transsarcolemmal Ca^2+^ entry via L-type Ca^2+^ channels and on intracellular store Ca^2+^ release. To test this hypothesis, we initially carried out gene expression and immunostaining studies to show that key Ca^2+^-handling proteins are expressed in hiPSC-CMs. To test for their functionality we then performed detailed laser-confocal Ca^2+^ imaging coupled with targeted pharmacological interventions. Initial studies confirmed the importance of transsarcolemmal Ca^2+^ entry through the L-type Ca^2+^ channels for modulation of the whole-cell [Ca^2+^]_i_ transients in these cells. We then demonstrated that hiPSC-CMs display functional and loaded RyR-regulated intracellular Ca^2+^ stores that contribute as well to the whole-cell [Ca^2+^]_i_ transient. In addition, we investigated the functionality of SR Ca^2+^ ATPase (SERCA) pumps, which serve as an important SR Ca^2+^ sequestration pathway. We found the SERCA pumps to be functional and responsible for the refilling of hiPSC-CMs' SR Ca^2+^ store content. Finally, we also present evidence showing the expression and functionality of inositol-1,4,5-trisphosphate receptors (IP3Rs) in hiPSC-CMs and demonstrate the important contribution of this alternative pathway to Ca^2+^-handling in these cells.

## Methods

### Differentiation of hiPSCs into cardiomyocytes

The hiPSC line utilized in the current study (hIH-1) was recently established in our laboratory [15] by retroviral delivery of three reprogramming factors: *OCT4*, *SOX2*, and *KLF4* together with valproic acid (VPA), a histone deacetylase inhibitor potentiating the reprogramming ability of these factors [16]. This hiPSCs line was demonstrated to fulfill all the criteria defining the iPSC state including full reprogramming, pluripotency, and genetic stability [15]. In the current study we used two clones (hIH-1 clones 1&2) of this line that were derived independently during reprogramming of the human fibroblasts. In addition, we also studied a second well-characterized hiPSCs line (hFib2-iPS cells; kindly provided by G.Q Daley) [8], [17], which was established by retroviral transduction of human fibroblasts with *OCT4*, *SOX2*, *c-MYC*, *KLF4*, together with hTERT and SV40-large T [17].

Undifferentiated hiPSC colonies were cultured on a mitotically-inactivated MEF feeder layer [8]. The culture medium consisted of 80%-knockout high-glucose glutamine-free DMEM with sodium-pyruvate supplemented with 20%-serum-replacement, 1 mM L-glutamine, 0.1 mM mercaptoethanol, 4 ng/mL human recombinant basic fibroblast-growth-factor, and 1%-nonessential amino acid stock (Invitrogen).

To induce differentiation, hiPSCs were dispersed into small clumps using collagenase-IV (Life-Technologies, 1 mg/mL, 20 min) and cultivated in suspension where they aggregated to form embryoid bodies (EBs). The EBs were plated after 10 days on gelatin-coated culture dishes and observed for the appearance of spontaneous contracting areas. The beating areas within the EBs were mechanically microdissected at 30–50 days following the appearance of spontaneous beating to allow comparison with studies assessing hESC-derived cardiomyocytes at similar developmental stages [18], [19]. This was followed by enzymatic dispersion (1 mg/ml collagenase B, Roche, Mannheim, Germany) at 37°C for 30 min to derive single cardiomyocytes or small monolayered clusters. After dispersal, the cells were plated onto fibronectin-coated glass coverslips for experimentation.

### Reverse transcription PCR (RT-PCR) analysis

The expression of various cardiac Ca^2+^-handling genes was tested using semi-quantitative RT-PCR. Total RNA was extracted from human embryonic kidney cells (HEK293, American Type Culture Collection (ATCC), Manassas, VA) and from beating hiPSC-CMs using RNeasy Mini Kit (Qiagen). cDNA was synthesized using Verso™-RT-PCR-Kit (Thermo-Scientific). 1.6 ng of cDNA was subjected to the following PCR program: 3 min at 93°C, 30 sec at 93°C, 30 sec at 60°C, and 30 sec at 72°C. The PCR-related primers are detailed in Table 1.

**Table 1:** Primers names and sequences.

Gene	Primer
Ca_V_1.2 S	TGACATCGAGGGAGAAAACT
Ca_V_1.2 A.S	ACATTAGACTTGACTGCGGC
RyR2 S	TAGATTTATAAGGGGCCTTG
RyR2 A.S	GATTCTTCAGGGCTCGTAGT
IP3R2 S	GAAGAAACTACAGCACGCTG
IP3R2 A.S	TTCTCCAGTAAAGCAGGTAA
SERCA2 S	GAGAACGCGCACACCAAGA
SERCA2 A.S	TTGGAGCCCCATCTCTCCTT
Calsequestrin S	ATAGAGTTTGATGGCGAGTT
Calsequestrin A.S	TGATGTAGTCTTCAATGCGT
Phospholamban S	CTGCCAAGGCTACCTAAAAG
Phospholamban A.S	AGCTGAGCGAGTGAGGTATT
β-Tubulin S	CCGGACAGTGTGGCAACCAGATCG
β-Tubulin A.S	TGGCCAAAAGGACCTGAGCGAACGG

Abbreviations: Ca_V_1.2, L-type Ca^2+^ channels; RyR2, ryanodine receptor; IP3R2, inositol 1,4,5-triphosphate receptor; SERCA2, SR Ca ATPase;.

### Immunocytochemistry

Single cells and small monolayered clusters were fixed using 4% paraformaldehyde and permeabilized with 1% Triton-X-100 (Sigma). Cells were blocked with 5% horse serum and 1% bovine serum albumin and incubated overnight at 4°C with primary antibodies targeting sarcomeric α-actinin (1∶200, Sigma-Aldrich), ryanodine receptor (1∶1,000, Chemicon), and pan-IP3R (reacting with the C-terminal cytoplasmic domain of IP3R types 1, 2, and 3) (1∶10, Millipore). The secondary antibodies were Cy3-conjugated donkey anti-mouse and Cy2-conjugated goat anti-rabbit IgG antibodies at 1∶200 for 1 h (Jackson). Nuclei were counterstained with DAPI (Sigma). Preparations were examined using a laser-scanning confocal microscope (Zeiss LSM-510-PASCAL).

### Ca^2+^ Imaging

Cells were loaded with 5 µM fluo-4 fluorescent Ca^2+^ indicator (Molecular Probes) in the presence of Pluronic F-127 (Molecular Probes) at a dilution of 2∶1 to allow the recording of intracellular Ca^2+^-transients (whole-cell [Ca^2+^]_i_ transients) as previously described [18]. Experiments were conducted in the presence of indicator-free tyrode solution containing in mM: NaCl-140, KCl-5.4, CaCl_2_-1.8, MgCl_2_-1, HEPES-10, glucose-10 at pH 7.4 via 1 mM NaOH. The tyrode solution was superfused at a rate of 1 ml/minute and at a temperature of 37°C. Intracellular Ca^2+^-transients were recorded using a confocal imaging system (Fluoview; Olympus) mounted on an upright BX51WI Olympus microscope equipped with a X60 water objective [20]. Data were analyzed utilizing MatLab-based custom-written software.

To investigate caffeine mobilization of store Ca^2+^, temporally-limited puffs of caffeine were applied. This specific technique [18] was chosen to overcome the technical issue of the rate of caffeine delivery to the cells. The caffeine puff (20 mM, <2s) was applied by pressure-ejection through a pipette situated approximately 100 µm away from the target cells' position. The focal puff pipette was positioned central to the plane of the line scan and in the direction of constant bulk flow of the tyrode solution.

### Statistical analysis

Data are presented as mean ± standard error of mean (SEM). Student's paired t-test was used to compare between means. When the effects of multiple concentrations of the pharmacological agents were studied, then one way ANOVA was used followed by Dunnet's post-hoc comparison to baseline values. p<0.05 was considered to be statistically significant.

## Results

### Expression of Ca^2+^-handling molecules in hiPSC-CMs

**Figure 1:**
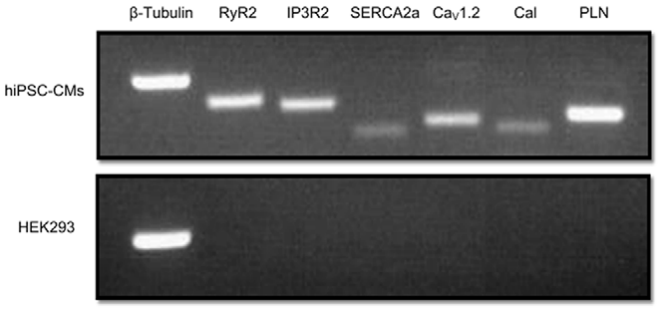
Semi-quantitative RT-PCR analysis for cardiac-specific Ca^2+^-handling proteins in hiPSC-CMs. Gene expression pattern of proteins involved in Ca^2+^-handling in hiPSC-CMs (upper panel) and control HEK293 cells. The lower panel displays genes expression in control HEK293 cells. β-tubulin was used as a normalizing house-keeping gene. Abbreviations: RyR2, ryanodine receptor; IP3R2, inositol 1,4,5-triphosphate receptor; SERCA2a, SR Ca ATPase; Ca_V_1.2, L-type Ca^2+^ channels; Cal, calsequestrin, and PLN, phospholamban.

To evaluate whether cardiac-related Ca^2+^-handling molecular components are present in the hiPSC-CMs, we first used semi-quantitative RT-PCR analysis. We assessed for the expression of the following Ca^2+^-handling proteins: RyR2, IP3R2, SERCA2a, Ca_v_1.2 (encoding the α-1C subunit of the L-type Ca^2+^ channel), Calsequestrin, and Phospholamban. As can be seen in Figure 1, all of these genes were expressed in the hiPSC-CMs while absent in control HEK293 cells.

### Spontaneous whole-cell [Ca^2+^]_i_ transients in hiPSC-CMs

**Figure 2:**
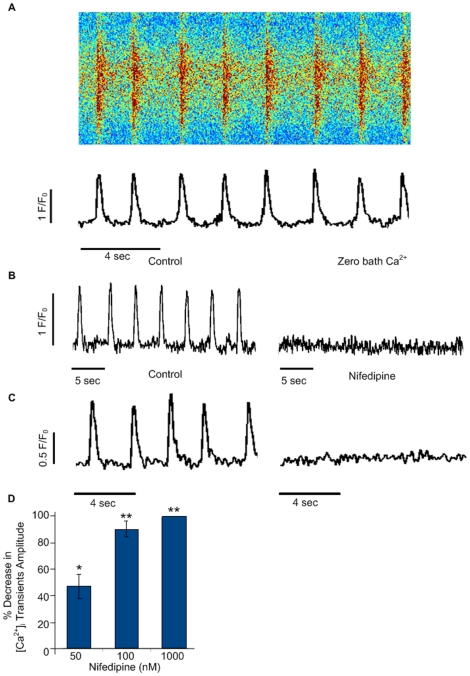
Whole-cell [Ca^2+^]_i_ transients in hiPSC-CMs and their requirement for Ca^2+^ influx via L-type Ca^2+^ channels. (**A**) Confocal line-scan images showing changes in intracellular Ca^2+^ in a fluo-4 loaded hiPSC-CM (derived from the hIH1 line). The images showing spontaneous whole-cell [Ca^2+^]_i_ transients are displayed either as a distance-time plot (upper panel) or as a line-scan tracing (lower panel). (**B**) Whole-cell [Ca^2+^]_i_ transients in the presence (left) and absence (right) of bath Ca^2+^. (**C**) Whole-cell [Ca^2+^]_i_ transients before (left) and after (right) application of nifedipine (1 µM). (**D**) Dose-response curve showing the percentage of decrease in [Ca^2+^]_i_ transients amplitude relative to baseline conditions as function of nifedipine concentration (0.05, 0.1 and 1 µM, n = 6). *p<0.05 and **p<0.01 when mean absolute values were compared to baseline values. Abbreviations: F/Fo, fluorescence (F) normalized to baseline fluorescence (Fo); sec, seconds.

Spontaneous whole-cell [Ca^2+^]_i_ transients in hiPSC-CMs were recorded from spontaneously beating dispersed single cells or small monolayered clusters, under native cytosolic conditions. These transients were monitored in Fluo-4 (a fluorescent Ca^2+^ indicator) loaded cells, examined under a laser scanning confocal microscope utilizing the line-scan mode. Line scans were adjusted to avoid the cell nuclei and were localized at mid-cell *z*-section depth. Under control conditions, in the presence of 1.8 mM external bath Ca^2+^, spontaneous whole-cell [Ca^2+^]_i_ transients were recorded as cell-wide rhythmic events in all cells tested (n = 96) (Figure 2A).

### Ca^2+^ influx via L-type Ca^2+^ channels contributes to whole-cell [Ca^2+^]_i_ transients

Transmembranal Ca^2+^ influx is an important initial trigger for excitation-contraction coupling in adult cardiomyocytes [9] and in hESC-derived cardiomyocytes (hESC-CMs) [19]. Therefore, the next step was to investigate whether the development of hiPSC-CMs whole-cell [Ca^2+^]_i_ transients require external Ca^2+^. To this end, we recorded whole-cell [Ca^2+^]_i_ transients in the presence (1.8 mM bath Ca^2+^) and absence of Ca^2+^ in the bath solution (n = 6). As can be appreciated in Figure 2B, in the absence of bath Ca^2+^ the whole-cell [Ca^2+^]_i_ transients were completely abolished.

To test whether the L-type Ca^2+^ channel serves as an important transmembranal Ca^2+^ influx pathway in hiPSC-CMs, as documented in adult cardiomyocytes [9], we tested the effect of Nifedipine, a L-type Ca^2+^ channel blocker. Whole-cell [Ca^2+^]_i_ transients were recorded before and after the application of 1 µM Nifedipine (Figure 2C). Similarly to what was observed in the absence of bath Ca^2+^ (Figure 2B), 1 µM Nifedipine led to the complete elimination of whole-cell [Ca^2+^]_i_ transients (n = 9). Dose-response studies using lower concentrations of nifedipine (0.05–1 µM, n = 6) demonstrated that the cells were extremely sensitive to L-type channel blockade with a steep decrease in [Ca^2+^]_i_ transients amplitude observed at a very low concentration (Figure 2D).

To verify that the results obtained were not due to clonal or line variations, we compared the results obtained in cardiomyocytes differentiated from two different clones of the main hiPSC line studied (hIH1 - clones 1&2 derived independently from the human fibroblasts) as well as from an additional well-characterized hiPSC line (hfib2-5) derived using the traditional 4-factors method [8], [17]. The dependency of whole-cell [Ca^2+^]_i_ transients on the presence of functional L-type Ca^2+^ channels was found to be independent of the specific hiPSC clone or line used. Thus, Nifedipine (1 µM) application resulted in complete elimination of whole-cell [Ca^2+^]_i_ transients in all cases (Figure S1).

Taken together these data confirm that transmembranal Ca^2+^ influx and specifically Ca^2+^ entry via L-type Ca^2+^ channels are important requirements for the generation of whole-cell [Ca^2+^]_i_ transients in hiPSC-CMs.

### Functional RyR-mediated intracellular Ca^2+^ stores exist and contribute to whole-cell [Ca^2+^]_i_ transients

**Figure 3:**
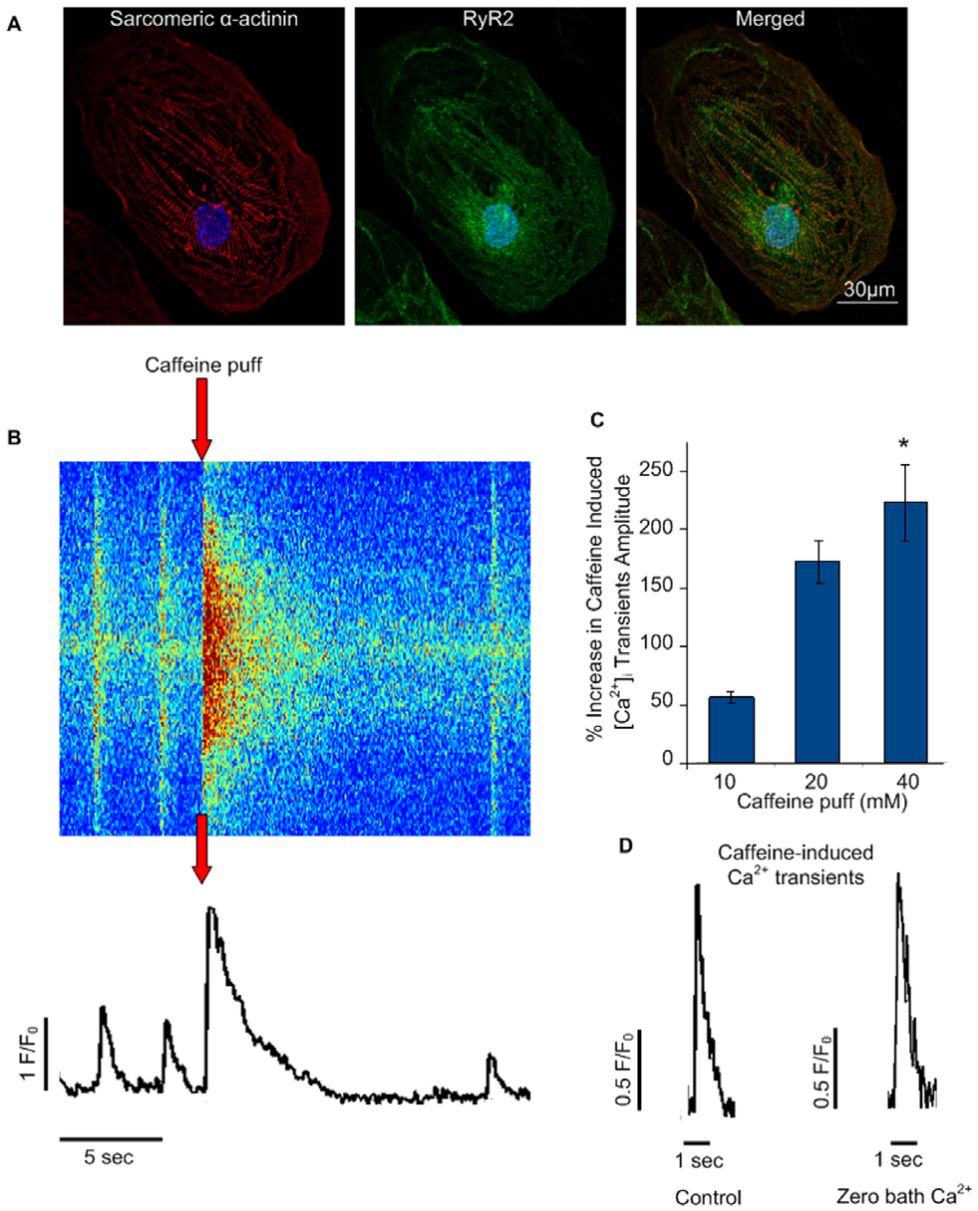
Localization and functionality of Ca^2+^ store ryanodine receptors. (**A**) A hIH1 hiPSC-CM co-labeled with antibodies for sarcomeric α-actinin (left) and RyR2 (middle). The merged image is displayed in the right panel. (**B**) A line-scan presenting the effect of 20 mM caffeine puff application (noted by the arrows). (**C**) A dose-response curve displaying the magnitude of the caffeine-induced Ca^2+^ release (quantified by the percentage increase of the amplitude of the caffeine-induced transient relative to the preceding action-potential induced [Ca^2+^]_i_ transient) as function of the caffeine bolus dosage (10, 20 and 40 mM). *p<0.05 when compared to the lowest (10 mM) dosage. (**D**) A line-scan tracings presenting caffeine-induced Ca^2+^ transients in the presence (left) and absence (right) of bath Ca^2+^. Abbreviations: F/Fo, fluorescence (F) normalized to baseline fluorescence (Fo); sec, seconds.

We next conducted immunocytostaining studies of hiPSC-CMs probing for both RyR2 and sarcomeric α-actinin in small monolayered clusters (Figure 3A). As previously shown in hESC-CMs [18], [21] sarcomeric α-actinin staining in hiPSC-CMs displayed a relatively disorganized striated sarcomeric arrangement (Figure 3A, left). RyR2 expression was exhibited throughout the cytosol (Figures 3A, middle), with some myofilaments co-localization (Figure 3A, right). The perinuclear region displayed intense staining as was similarly observed in mouse ESC-CMs [22] and hESC-CMs [18].

To determine whether hiPSC-CMs possess loaded SR Ca^2+^ stores that release Ca^2+^ via functional RyRs we tested for caffeine responsiveness. Caffeine mobilization of store Ca^2+^ and its effect on whole-cell [Ca^2+^]_i_ transients were measured by applying a pressure-ejected caffeine puff (20 mM, 2 sec) on to fluo-4 loaded hiPSC-CMs. As can be observed in Figure 3B, caffeine application elicited an instantaneous, rapid, and large release of Ca^2+^ from the intracellular stores, resulting in a high amplitude caffeine-induced Ca^2+^ transient (n = 26). This was followed by reversible quiescence of whole-cell [Ca^2+^]_i_ transients (Fig. 3B) postulated to be a consequence of intracellular Ca^2+^ stores depletion. This phenomenon was noted in cardiomyocytes derived from all hiPSCs clones and lines studied (Figure S2A). Finally, dose-response studies (10, 20, and 40 mM caffeine puffs, n = 6) showed an escalating effect with an increase in the relative magnitude of caffeine-induced Ca^2+^ release (Figure 3C).

Next, it was important to validate that the caffeine-induced [Ca^2+^]_i_ transient was indeed a consequence of RyR-mediated SR Ca^2+^ release. To exclude the plausible contribution of Ca^2+^ influx through voltage-gated Ca^2+^ channels 20 mM caffeine puffs were applied in the presence (1.8 mM Ca^2+^; Figure 3D, left) and absence (Figure 3D, right) of bath Ca^2+^. Similarly to what was observed under control conditions (Figure 3D, left), caffeine puffs applied in the absence of bath Ca^2+^ (n = 6) induced an instantaneous rapid caffeine-induced [Ca^2+^]_i_ transient (Figure 3D, right) displaying an amplitude similar to that observed under control conditions. To exclude the possibility that the caffeine-induced [Ca^2+^]_i_ transient was a result of a mechanical stimulation to the cell surface, caused by the actual pressure injected puff, control puff (tyrode solution) trials were conducted. These control puffs did not trigger any apparent intracellular Ca^2+^ response (data not shown).

**Figure 4:**
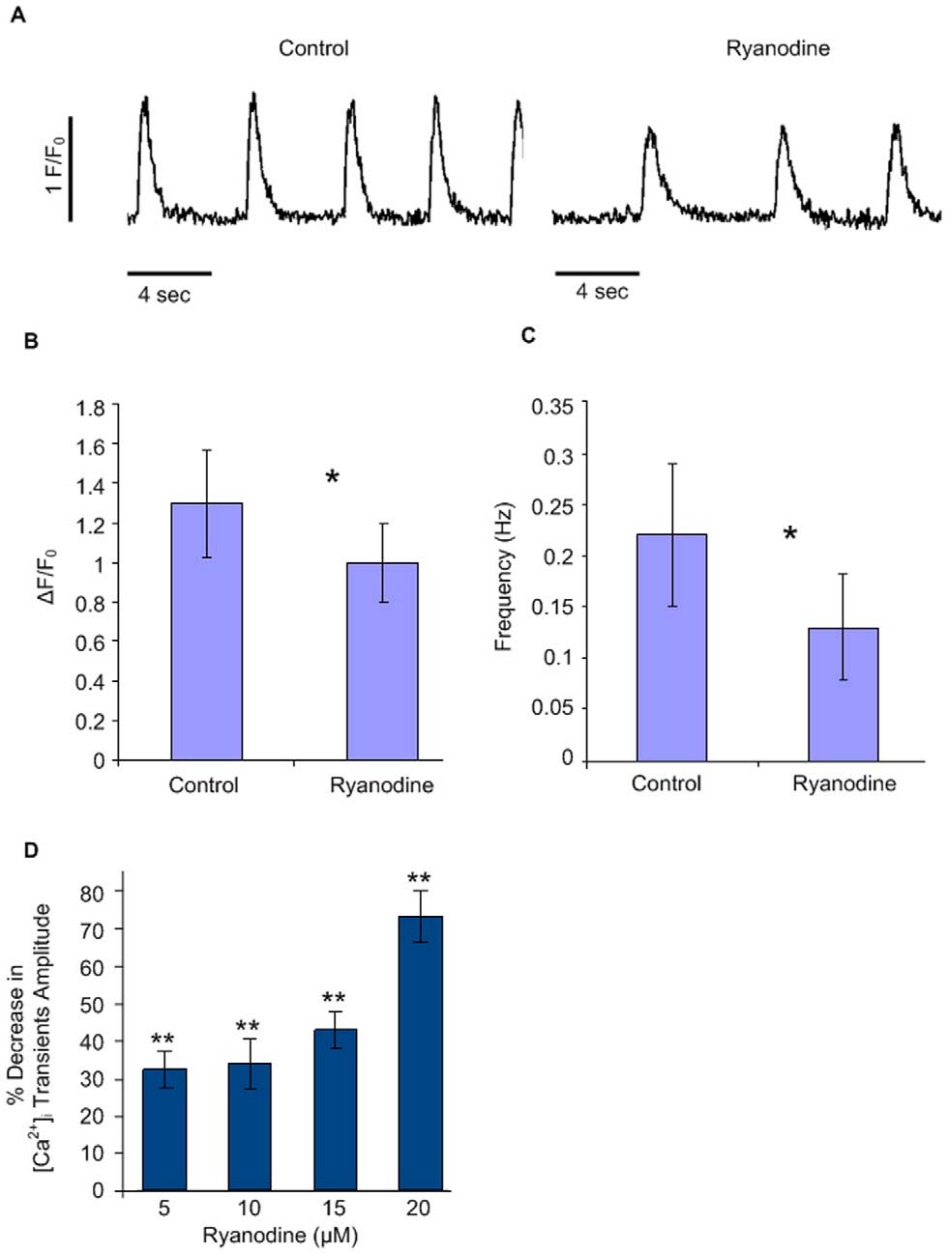
Ryanodine-sensitive Ca^2+^ stores. (**A**) Line-scan tracings of whole-cell [Ca^2+^]_i_ transients recorded from a representative hIH1 hiPSC-CM under control conditions (left) and in the presence of 10 µM Ryanodine (right). (**B–C**) Summary of the ryanodine-mediated (10 µM) diminution in whole-cell [Ca^2+^]_i_ transients amplitude (B) and frequency (C). *p<0.05 when compared to baseline (control) values. (**D**) Dose-response curve for the effects of ryanodine (5, 10, 15 and 20 µM) on whole-cell [Ca^2+^]_i_ transients amplitude displayed as the percentage of decrease from baseline values. **p<0.01 when mean absolute values were compared to baseline values. Abbreviations: F/Fo, fluorescence (F) normalized to baseline fluorescence (Fo); sec, seconds.

Finally, we also tested the effect of ryanodine, a RyR antagonist. For this purpose we monitored whole-cell [Ca^2+^]_i_ transients before (Figure 4A, left) and after (Figure 4A, right) application of ryanodine (10 µM). Ryanodine administration led to a significant reduction in Ca^2+^ release, as observed by the decrease in whole-cell [Ca^2+^]_i_ transients amplitude (Figure 4B; n = 14; p<0.05) and also to significant slowing of whole-cell [Ca^2+^]_i_ transients frequency (Figure 4C; n = 6; p<0.05). The effect of ryanodine was noted in cardiomyocytes derived from all hiPSC clones and lines studied (Figure S2B) and was dose-dependent, as increasing doses of ryanodine (5, 10, 15, and 20 µM, n = 8) led to a gradual decrease in whole-cell [Ca^2+^]_i_ transients amplitude in both lines studied (Figures 4D and S2C). Taken together, these data demonstrate that hiPSC-CMs display caffeine-responsive and ryanodine-sensitive SR Ca^2+^ stores capable of unloading Ca^2+^ via RyR-mediated Ca^2+^ release and contributing to whole-cell [Ca^2+^]_i_ transients.

### SERCA-mediated SR Ca^2+^ uptake is required for whole-cell [Ca^2+^]_i_ transients

**Figure 5:**
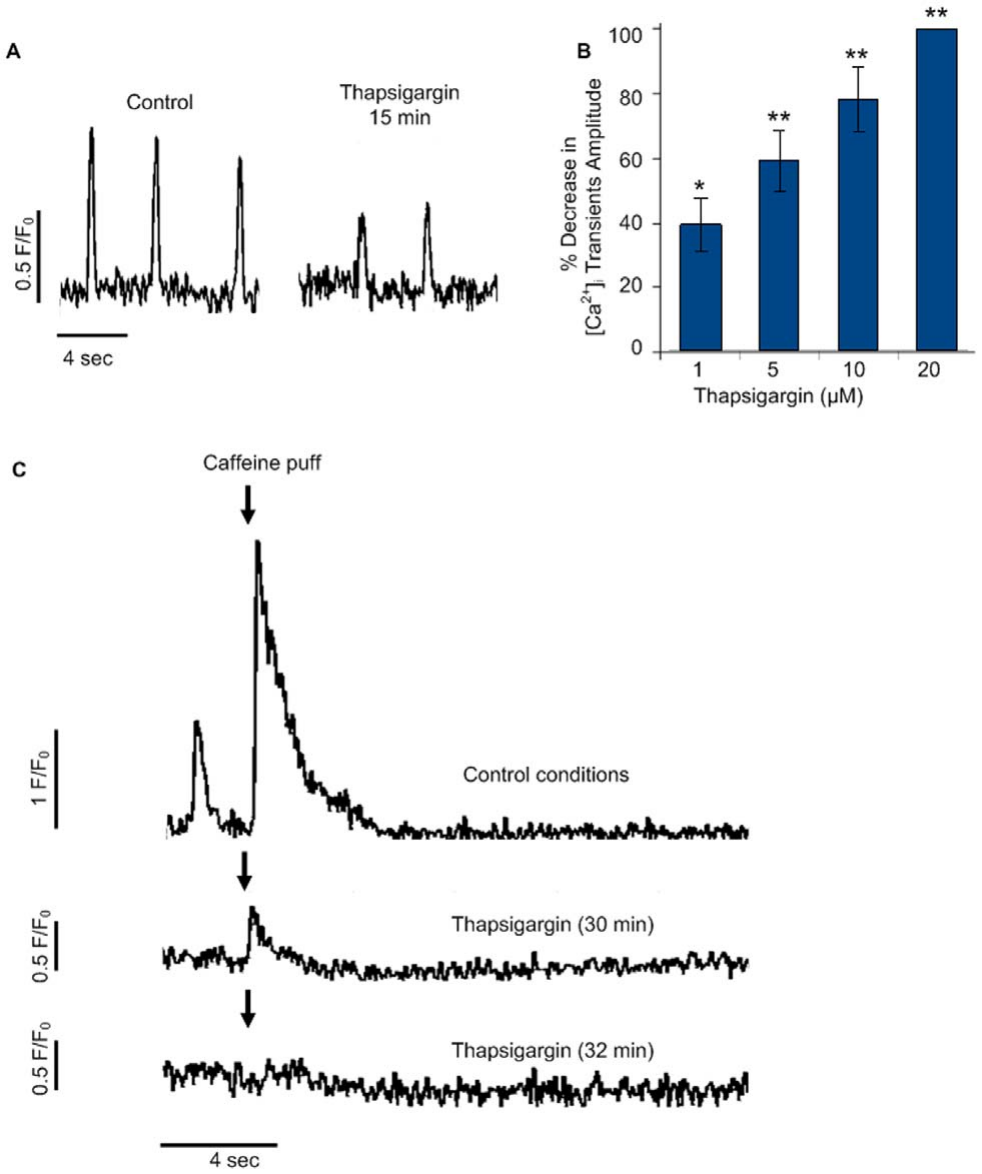
The effects of SERCA inhibition. (**A**) Line-scan tracings of whole-cell [Ca^2+^]_i_ transients in a hIH1 hiPSC-CM under control conditions (left) and after 15 minutes (right) of continuous 10 µM thapsigargin exposure. (**B**) Dose-response effects of thapsigargin (1, 5, 10 and 20 µM) on [Ca^2+^]_i_ transients amplitude (displayed as percentage decrease versus baseline values). *p<0.05 and **p<0.01 when mean absolute values were compared to baseline values. (**C**) Caffeine-induced Ca^2+^ transients (denoted by arrows) under control conditions (upper), after 30 minutes (middle), and 32 minutes (lower) of thapsigargin exposure. Abbreviations: F/Fo, fluorescence (F) normalized to baseline fluorescence (Fo); sec, seconds.

We next tested for the functionality and contribution of another important Ca^2+^-handling protein situated on the SR membrane, SERCA. To test for SERCA functionality in hiPSC-CMs we recorded whole-cell [Ca^2+^]_i_ transients before (Figure 5A, left) and after (Figure 5A, right panel) application of 10 µM thapsigargin, a specific SERCA inhibitor. Application of 10 µM thapsigargin (15 min incubation) resulted in a decrease in whole-cell [Ca^2+^]_i_ transients' amplitude in both lines studied (n = 7, Figure 5A, and Figure S3A). The effect of thapsigargin was dose-related with a greater decrease in whole-cell [Ca^2+^]_i_ transients amplitude (observed at 15 min following drug application) with escalating doses (1, 5, 10 and 20 µM, Figure 5B).

We have shown above (Figures 3, 4, 5A) that in the case of hiPSC-CMs SR Ca^2+^ release is an important contributor of whole-cell [Ca^2+^]_i_ transients. Therefore, we wanted to confirm whether the inhibiting effect of thapsigargin on whole-cell [Ca^2+^]_i_ transients was due to a decrease in SR Ca^2+^ content, as a consequence of SERCA Ca^2+^ uptake inhibition. To this end we conducted repeated SR Ca^2+^ load measurements by applying 20 mM caffeine puffs under control conditions (Figure 5C, upper panel) and in the presence of 10 µM thapsigargin (Figure 5C, middle and lower panels). In contrast, to the enhanced caffeine-induced [Ca^2+^]_i_ transient observed under control condition (Figure 5C, upper panel), application of a caffeine puff, when complete succession of all whole-cell [Ca^2+^]_i_ transients has taken place as a result of the thapsigargin uptake inhibition, produced only a minor effect. This effect was displayed as a miniscule caffeine-induced [Ca^2+^]_i_ transient (Figure 5C, middle panel), that was completely omitted in the subsequent caffeine puff (n = 7; Figure 5C, lower panel). A similar phenomenon was also observed in cardiomyocytes derived from a second hiPSC line (hfib2-5, Figure S3B). The absent caffeine-induced signal at this stage is postulated to be a consequence of the inability of the SR to reload due to SERCA uptake inhibition by thapsigargin.

### IP3-mediated calcium release contributes to whole-cell [Ca^2+^]_i_ transients

**Figure 6:**
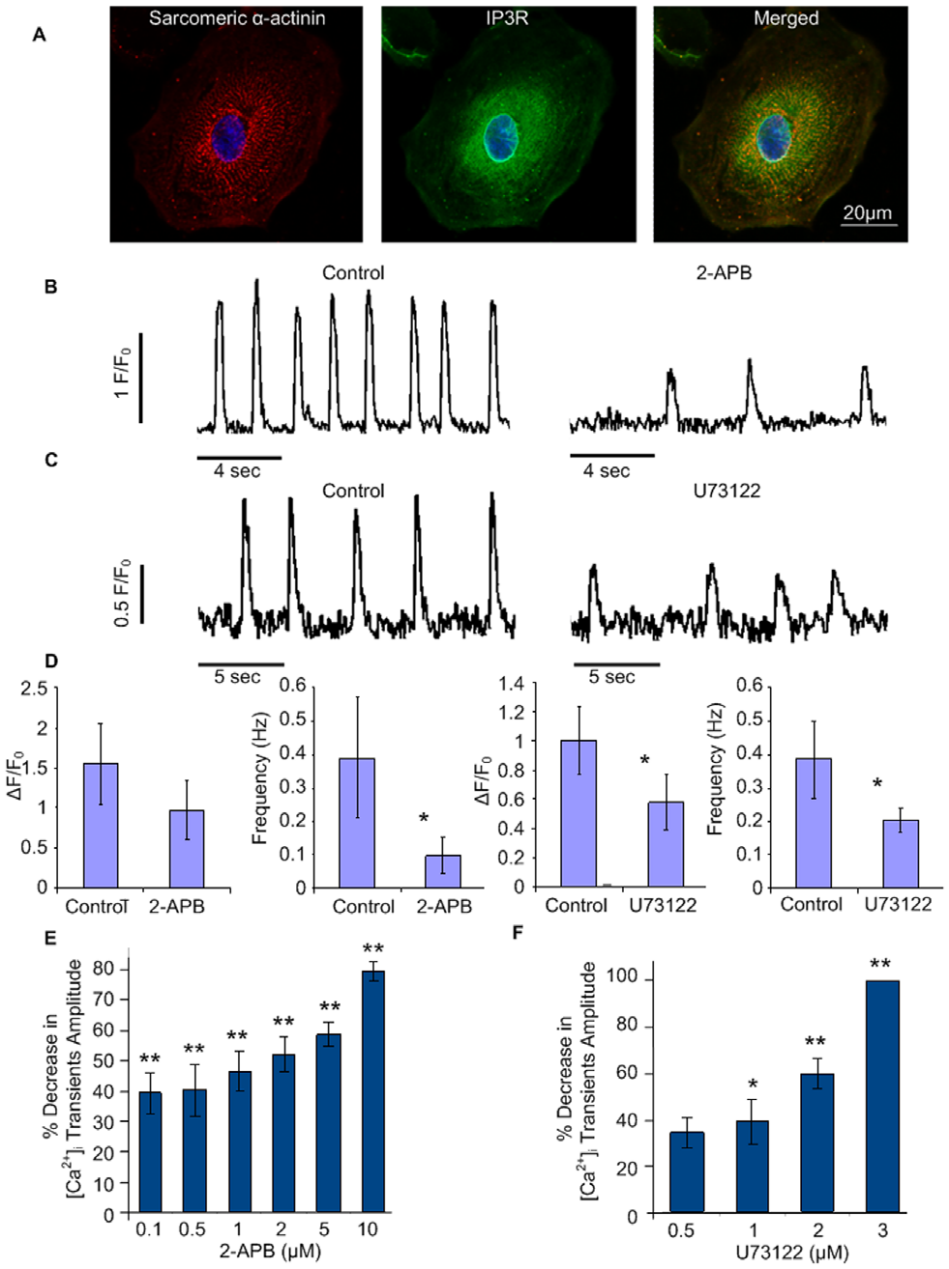
IP3Rs localization and function. (**A**) A hIH1 hiPSC-CM co-stained for pan-IP3R (middle) and sarcomeric α-actinin (left). Merged images are presented in the right panel. (**B**) Whole-cell [Ca^2+^]_i_ transients before (left) and after (right) 2-APB (2 µM) application. (**C**) Whole-cell [Ca^2+^]_i_ transients before (left) and after (right) U73122 (2 µM) application. (**D**) Summary of the effects of 2-APB and U73122 on whole-cell [Ca^2+^]_i_ transients amplitude and frequency. *p<0.05 when compared to baseline values. (**E–F**) Dose-response curves displaying the effects of 2-APB (0.1, 0.5, 1, 2, 5 and 10 µM) (E) and U73122 (0.5, 1, 2 and 3 µM) (F) on whole-cell [Ca^2+^]_i_ transients amplitude (displayed as the relative decrease (%) versus baseline values). *p<0.05 and **p<0.01 when mean absolute values were compared to baseline values. Abbreviations: F/Fo, fluorescence (F) normalized to baseline fluorescence (Fo); IP3R, inositol-1,4,5-trisphosphate receptor; sec, seconds.

IP3-dependent signaling has been shown to play an important role during the process of cardiac development [23], [24]. It was recently demonstrated that in both mouse [25], [26] and human [18], [27] ESC-CMs an IP3-releasable Ca^2+^ pool is expressed and functional. To evaluate the potential role of an IP3-releasable Ca^2+^ pool in hiPSC-CMs, we first utilized immunocytostaining to detect the presence of the IP3R. These stainings displayed positive IP3R immunosignal, strongly distributed around the nucleus (Figure 6A), in a similar fashion to that observed in neonatal rat cardiomyocytes [28], mouse ESC-CMs [26], and hESC-CMs [18].

To assess the potential contribution of the IP3R to the modulation of whole-cell [Ca^2+^]_i_ transients in hiPSC-CMs, we tested the effect of IP3R blockade utilizing two different antagonistic approaches. First, whole-cell [Ca^2+^]_i_ transients were recorded before (Figure 6B, left) and after (Figure 6B, right) application of a low concentration of 2-aminoethoxyphenyl borate (2-APB, 2 µM, n = 14), a well-known cell-permeate IP3R antagonist. 2-APB application resulted in a significant decrease in whole-cell [Ca^2+^]_i_ transients amplitude (Figures 6B,D) and significantly slowed down whole-cell [Ca^2+^]_i_ transients frequency (Figure 6B,D). This effect was also dose-related (0.1, 0.5, 1, 2, 5, and 10 µM; n = 8) as displayed in Figure 6E. Strengthening the above-mentioned results, application of 2 µM of U73122 (a phosopholipase C inhibitor, n = 15) led to a significant diminution of whole-cell [Ca^2+^]_i_ transients amplitude (Figure 6C–D) and a slowing of whole-cell [Ca^2+^]_i_ transients frequency (Figure 6C–D). This effect was dose-dependent (0.5, 1, 2, and 3 µM; n = 8; Figure 6E). The effects of 2-APB and U73122 were found to be independent of the hiPSC line used (Figure 6 and Figure S4). These observations imply that in hiPSC-CMs an IP3-releasable Ca^2+^ pool is functional and contributes to the modulation of Ca^2+^-handling in these cells.

## Discussion

The ability to derive hiPSCs by reprogramming of adult human fibroblasts [2], [3] coupled with the capability to coax the differentiation of the generated hiPSCs into the cardiac lineage [6], [7], [8] opens unique opportunities for basic and translational cardiovascular research. Yet, to fulfill the promise of this unique technology in areas such as cardiovascular regenerative and personalized medicine, it is important that the generated hiPSC-CMs express a cardiomyocyte-specific phenotype; possessing, among other requirements, functional excitation-contraction coupling components.

In the current study, we focused on studying the Ca^2+^-handling properties of hiPSC-CMs. Our major findings consist of the following: (1) Mature molecular components RyR2, IP3R2, SERCA2a, Ca_V_1.2, calsequestrin, and phospholamban are expressed in these cells; (2) hiPSC-CMs display spontaneous whole-cell [Ca^2+^]_i_ transients; (3) Ca^2+^ entry via L-type Ca^2+^ channels is required for triggering whole-cell [Ca^2+^]_i_ transients; (4) Caffeine-responsive and ryanodine-sensitive Ca^2+^ stores are loaded and functional; (5) RyR-mediated SR Ca^2+^ release contributes to whole-cell [Ca^2+^]_i_ transients; (6) SERCA pumps are functional and enable the refilling of SR Ca^2+^ content, required for the modulation of whole-cell [Ca^2+^]_i_ transients; (7) An IP3-releasable Ca^2+^ pool is expressed, functional, and contributes to whole-cell [Ca^2+^]_i_ transients; and (8) the results obtained are comparable in cardiomyocytes derived from different differentiation experiments of the same hiPSC line, from different hiPSC clones (derived independently from fibroblasts of the same individual), and from different hiPSCs lines established using different techniques (using either three or four reprogramming factors). Taken together we conclude that whole-cell [Ca^2+^]_i_ transients in hiPSC-CMs depend on both Ca^2+^ influx via L-type Ca^2+^ channels and intracellular Ca^2+^ store release, as previously documented in mouse [25], [26] and human ESC-CMs [18], [19].

### Whole-cell [Ca^2+^]_i_ transients in hiPSC-CMs depend on Ca^2+^ entry via L-type Ca^2+^ channels and Ca^2+^ release from RyR-mediated SR Ca^2+^ stores

In adult cardiomyocytes the development of an action potential triggers the opening of L-type Ca^2+^ channels. The resulting Ca^2+^ influx serves as a trigger for activating RyR-mediated SR Ca^2+^ release by a mechanism known as CICR [9], [10]. In addition to the well-established CICR mechanism in adult mammalian cardiomyocytes other mechanistic models were also proposed to be responsible for E-C coupling in various species and at earlier cardiomyocyte developmental stages. These include reports in frog [13] and turtle [11] adult ventricular cells as well as in primary embryonic murine myocytes [12], in which whole-cell [Ca^2+^]_i_ transients were shown to be derived solely from Ca^2+^ influx through membrane Ca^2+^ channels. In contrast, global whole-cell [Ca^2+^]_i_ transients in early cardiac cells derived from mouse ESC [14] were reported to be the result of spontaneous Ca^2+^ release from intracellular Ca^2+^ stores without the triggering of membrane Ca^2+^ currents.

The mechanism underlying E-C coupling in hESC-CMs is somewhat controversial. While some reports suggested the absence of a functional SR Ca^2+^ store in hESC-CMs and postulated that essentially all of the [Ca^2+^]_i_ transients in these cells were a consequence of transsarcolemmal Ca^2+^ influx via membranal Ca^2+^ channels [29], others have argued for a more mature-like CICR mechanism [18], [19], [30]. The latter studies reported the presence of a functional caffeine-responsive and ryanodine-sensitive SR Ca^2+^ store in at least a subset [30] if not all [18], [19], of the cells tested; in a similar manner to the hiPSC-CMs studied in the current study.

Our results support the contribution of both the transsarcolemmal Ca^2+^ influx and intracellular Ca^2+^ store release to whole-cell [Ca^2+^]_i_ transients in hiPSC-CMs. The importance of the L-type Ca^2+^ current in generating whole-cell [Ca^2+^]_i_ transients in these cells was manifested by the elimination of these transients in the absence of external Ca^2+^ or in the presence of nifedipine, a selective L-type Ca^2+^ channel antagonist. A similar requirement for external Ca^2+^ and the consequent transsarcolemmal Ca^2+^ influx was documented in adult cardiomyocytes [9], [31], [32], hESC-CMs [19] and mouse ESC-CMs [33], [34].

The contribution of Ca^2+^ release from intracellular SR Ca^2+^ stores to whole-cell [Ca^2+^]_i_ transients was demonstrated by pharmacological studies interfering either with SR Ca^2+^ release (caffeine and ryanodine) or reuptake (thapsigargin). Caffeine increases RyR2s opening, leading to a single large-amplitude caffeine-induced Ca^2+^ transient, considered to be a descriptive index of the level of SR Ca^2+^ load [35]. In hiPSC-CMs a local pressure ejected puff of caffeine elicited a local bolus release of Ca^2+^, followed by a short and reversible succession of whole-cell [Ca^2+^]_i_ transients. These results suggest that caffeine induced depletion of the SR Ca^2+^ store and point to a whole-cell [Ca^2+^]_i_ transient dependency on SR Ca^2+^ content.

The main Ca^2+^ source for the caffeine induced [Ca^2+^]i increase is RyR-mediated SR Ca^2+^ release and is not dominated by Ca^2+^ influx via voltage-gated Ca^2+^ channels. This was demonstrated by the similar caffeine-induced rise in intracellular Ca^2+^ documented in the absence of extracellular bath Ca^2+^ further confirming the presence of a caffeine-responsive intracellular Ca^2+^ store. Similar results were also acquired in hESC-CMs [18], [19].

We also applied the RyR antagonist, ryanodine, to further study hiPSC-CMs RyR-mediated SR Ca^2+^ release. Ryanodine, has been reported to reduce by approximately twofold the conductance of RyRs in the SR [36]. In hiPSC-CMs, ryanodine application led to a dose-dependent diminution in Ca^2+^ release observed as a significant decrease in the amplitude of whole-cell [Ca^2+^]i transients. A similar ryanodine induced effect was also reported in pacemaker cells isolated from rabbit sinoatrial node [37], in ESC-CMs [18], [30] and in mouse ESC-CMs [33]. In addition, in hiPSC-CMs ryanodine application also resulted in slowing of the spontaneous whole-cell [Ca^2+^]i transients firing rate. This phenomenon was also previously documented in rabbit sinoatrial node pacemaker cells where a similar slowing in firing rate was detected in the presence of ryanodine [37].

### Functional SERCA pumps enable the loading of SR Ca^2+^ store content required for whole-cell [Ca^2+^]_i_ transients

For cellular relaxation to take place Ca^2+^ must be removed from the cytosol. In adult cardiomyocytes, one of the main Ca^2+^ removal pathways is the SR Ca^2+^ ATPase pump (SERCA) [9], [38]. These pumps decrease intracellular Ca^2+^, by sequestering Ca^2+^ back into the SR, and in this manner also regulate SR Ca^2+^ load [38]. In adult human cardiomyocytes, SERCA pumping activity is responsible for 70% of Ca^2+^ sequestration from the cytosol back into the SR [9]. To investigate the functionality and contribution of the SERCA pumps to whole-cell [Ca^2+^]i transients through their ability to reload the SR Ca^2+^ stores in hiPSC-CMs we applied the SERCA inhibitor thapsigargin. Thapsigargin acted slowly to progressively decrease the amplitude of whole-cell [Ca^2+^]i transients, eventually leading to their complete inhibition. A similar effect was observed in spontaneously beating fluo-4 loaded isolated mouse ESC-CMs^34^. An antagonistic effect of thapsigargin on [Ca^2+^]i transients was also reported in human ESC-CMs [19], [30].

The key role of SERCA in reloading the SR, and thereby indirectly modulating hiPSC-CMs whole-cell [Ca^2+^]_i_ transients, was further demonstrated by the miniscule effect of caffeine in hiPSC-CMs pretreated with thapsigargin, as a result of a pronounced diminution in SR Ca^2+^ content. Interestingly, under conditions of SERCA uptake inhibition (thapsigargin) a low SR Ca^2+^ content was retained yet [Ca^2+^]_i_ transients were completely abolished. This can be explained by reports showing that decrease in SR Ca^2+^ content can disproportionately inhibit SR Ca^2+^ release [39], which as shown here is an important contributor to hiPSC-CMs whole-cell [Ca^2+^]_i_ transients. In an immediate subsequent caffeine puff the caffeine-induced [Ca^2+^]i transient was completely omitted. The absent caffeine-induced signal at this stage is postulated to be a consequence of caffeine induced depletion of the SR Ca^2+^ store and the inability of the SR to accumulate Ca^2+^ as a result of the thapsigargin treatment.

### IP3R expression, function, and contribution to whole-cell [Ca^2+^]_i_ transients in hiPSC-CMs

IP3-mediated Ca^2+^ release presents a fundamental pathway for intracellular Ca^2+^ release in electrically non-excitable adult cells [40]. While, in adult cardiomyocytes IP3Rs contribution to cardiac physiology has remained elusive and controversial [9], [41], [42] they have been shown to play an important role during the process of cardiac development [23], [24], [42]. In fact, in the embryo the IP3R is reported to be the first expressed Ca^2+^ release channel [24], [42].

The IP3Rs have been reported to contribute to spontaneous activity in mouse ESC-CMs [25], [26] and are expressed and functional in hESC-CMs [18], [27]. To test for functionality and a potential contribution of an IP3-releasable Ca^2+^ pool to the modulation of Ca^2+^-handling in hiPSC-CMs we first examined the expression and localization of the IP3R at the protein level. Immunostainings of these hiPSC-CMs stained positive for IP3R with a strong subcellular distribution of the immunosignal around the nucleus (Figure 6A) in a similar manner to that observed in hESC-CMs [18], mouse ESC-CMs [26], and neonatal rat cardiomyocytes [28].

Next, to evaluate for IP3-releasable Ca^2+^ pool functionality and participation in the regulation of Ca^2+^-handling in hiPSC-CMs we tested the effect of IP3R blockade utilizing two different antagonistic approaches. First, to block IP3Rs we used the potent cell-permeate inhibitor 2-aminoethoxydiphenyl borate (2-APB) [25]. Application of 2-APB resulted in a significant dose-dependent diminution of whole-cell [Ca^2+^]_i_ transients amplitude, as was also reported in human ESC-CMs under these conditions [18]. In addition, a slowing of whole-cell [Ca^2+^]_i_ transients frequency was observed under the influence of 2-APB. Next we applied U73122, a phosopholipase C blocker (PLC). Blocking the activation of PLC inhibits a receptor-stimulated increase in the production of the second messenger IP3 required as a trigger for IP3R-mediated Ca^2+^ release [25], [27]. Superfusion of hiPSC-CMs with U73122 also significantly decreased whole-cell [Ca^2+^]_i_ transients amplitude and frequency. A U73122 PLC inhibitory effect was also reported in mouse ESC-CMs [25]. These observations imply that an IP3-releasable Ca^2+^ pool is expressed and functional in hiPSC-CMs and that the resulting IP3R-mediated Ca^2+^ release contributes to the modulation of Ca^2+^-handling of these cells.

### Potential clinical and research applications

The hiPSC technology has raised significant excitement with regards to its unique potential for regenerative medicine and for the study of various genetic disorders as well as for drug discovery and screening. In the current work we focused on the characterization of the Ca^2+^-handling properties of cardiomyocytes differentiated from hiPSCs and demonstrated that they share components that are present in adult cardiomyocytes, such as functional RyR-mediated SERCA-sequestering SR Ca^2+^ stores. Importantly, the results of this study showing similar properties in cardiomyocytes derived from different differentiation batches, from different hiPSCs clones, and from different hiPSCs lines may have important implications for their potential use for the aforementioned tasks. The hiPSC-CMs may serve as attractive cell-candidates for myocardial cell replacement therapy because of their inherent cardiac-specific properties and the potential for autologous therapy. Nevertheless, since functional compatibility between donor hiPSC-CMs and host myocardium is likely to contribute to an improved functional outcome of the cell engraftment as well as a reduction in potential pro-arrhythmic risk, detailed characterization of their Ca^2+^-handling characteristics is mandatory.

Similarly, phenotypic characterization of Ca^2+^-handling in these cells may be important if one wishes to utilize the iPSC technology for establishment of personalized *in-vitro* models of cardiac tissue for the development and testing of pharmacological compounds targeting these functional properties. Finally, detailed understanding of the Ca^2+^-handling properties of hiPSC-CMs generated from healthy individuals may be used as a future reference when studying E-C coupling in hiPSC-CMs derived from patients with genetic disorders involving Ca^2+^-handling. Such patient/disease-specific models can be established, for example, from families suffering from catecholinergic polymorphic ventricular tachycardia (CPVT) [43], [44], a potentially lethal disorder resulting from mutations in either the ryanodine receptor or calsequestrin.

### Conclusion

In the present work we investigated basic Ca^2+^-handling components of hiPSC-CMs. Our results show that hiPSC-CMs display functional and loaded RyR-regulated intracellular Ca^2+^ stores. These stores can release Ca^2+^ via RyRs and can reload their content through SR Ca^2+^ uptake utilizing functional SERCA pumps. We present evidence showing the expression and functionality of inositol-1,4,5-trisphosphate receptors (IP3Rs). Furthermore, our findings demonstrate that the observed whole-cell [Ca^2+^]_i_ transients in hiPSC-CMs depend on both sarcolemmal Ca^2+^ entry via L-type Ca^2+^ channels and on intracellular store Ca^2+^ release. Taken together hiPSC-CMs recapitulate functional key Ca^2+^ handling proteins that have been shown to be expressed and functional in mouse ESC-CMs [25], [26], [33], hESC-CMs [18], [19], [30], and adult cardiac tissue [9]. The results of the current study may have important implications for the potential applications of the iPSC technology in basic and translational cardiac research.

## Supporting Information

Figure S1 **Whole-cell [Ca^2+^]_i_ transients' requirement for Ca^2+^ influx via L-type Ca^2+^ channels as observed in cardiomyocytes derived from different hiPSCs clones and lines.** Whole-cell [Ca^2+^]_i_ transients recorded from (A): hIH1 clone 1, (B): hIH1 clone 2 and (C): hfib2-5 before (left) and after (right) application of nifedipine (1 µM). Abbreviations: F/Fo, fluorescence (F) normalized to baseline fluorescence (Fo); sec, seconds.
(PDF)

Figure S2 **Caffeine and Ryanodine-sensitive Ca^2+^ stores as displayed in cardiomyocytes derived from different hiPSCs clones and lines.** (**A**) A line-scan presenting the effect of 20 mM caffeine puff application (noted by the arrows) in hIH1 clone 1 (left), hIH1 clone 2 (middle) and hfib2-5 (right). (**B**) Line-scan tracings of whole-cell [Ca^2+^]_i_ transients recorded from hIH1 clone 1 (top), hIH1 clone 2 (middle) and hfib2-5 (bottom) under baseline conditions (left) and in the presence of 10 µM Ryanodine (right). (**C**) Dose-response curve for ryanodine (5, 10, and 20 µM) displayed as the percent decrease in [Ca^2+^]_i_ transients amplitude from baseline values (n = 5, **p<0.01 when mean absolute values were compared to baseline values). Abbreviations: F/Fo, fluorescence (F) normalized to baseline fluorescence (Fo); sec, seconds.
(PDF)

Figure S3 **The effects of SERCA inhibition in cardiomyocytes derived from the hfib2-5 line.** (**A**) Line-scan tracings of whole-cell [Ca^2+^]_i_ transients in a representative hfib2-5 hiPSC-CM under baseline conditions (left) and after 30 minutes (right) of constant 10 µM thapsigargin exposure. (**B**) Caffeine-induced Ca^2+^ transients (denoted by arrows) under baseline conditions (upper), after 30 minutes (middle), and 32 minutes (lower) of thapsigargin exposure. Abbreviations: F/Fo, fluorescence (F) normalized to baseline fluorescence (Fo); sec, seconds.
(PDF)

Figure S4 **The effect of IP3R inhibition in cardiomyocytes derived from the hfib2-5 line.** Whole-cell [Ca^2+^]_i_ transients in representative hfib2-5 hiPSC-CMs before (left) and after (right) 2-APB (2 µM) application (A) and before (left) and after (right) U73122 (2 µM) application (B). Abbreviations: F/Fo, fluorescence (F) normalized to baseline fluorescence (Fo); IP3R, inositol-1,4,5-trisphosphate receptor; sec, seconds.
(PDF)
