# Supplementary figures and images for: Calcium Handling in Human Induced Pluripotent Stem Cell Derived Cardiomyocytes
Source: PLoS One. 2011 Apr 1;6(4):e18037. doi: 10.1371/journal.pone.0018037 (PMC3069979; doi:10.1371/journal.pone.0018037)

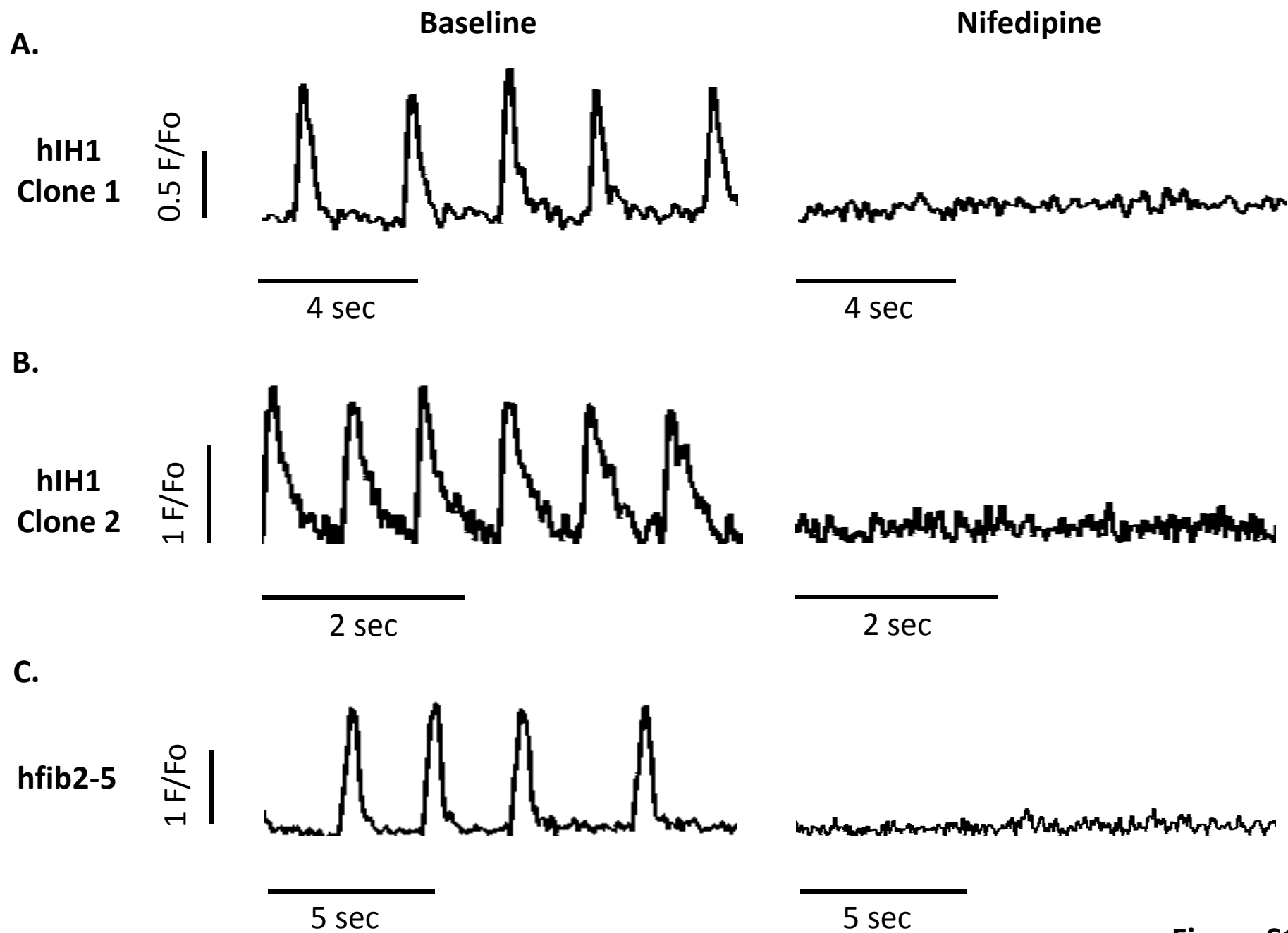

**Figure S1**

Supplement: Figure S1 — Whole-cell [Ca2+]i transients' requirement for Ca2+ influx via L-type Ca2+ channels as observed in cardiomyocytes derived from different hiPSCs clones and lines. Whole-cell [Ca2+]i transients recorded from (A): hIH1 clone 1, (B): hIH1 clone 2 and (C): hfib2-5 before (left) and after (right) application of nifedipine (1 µM). Abbreviations: F/Fo, fluorescence (F) normalized to baseline fluorescence (Fo); sec, seconds. (PDF) [file pone.0018037.s001.pdf]

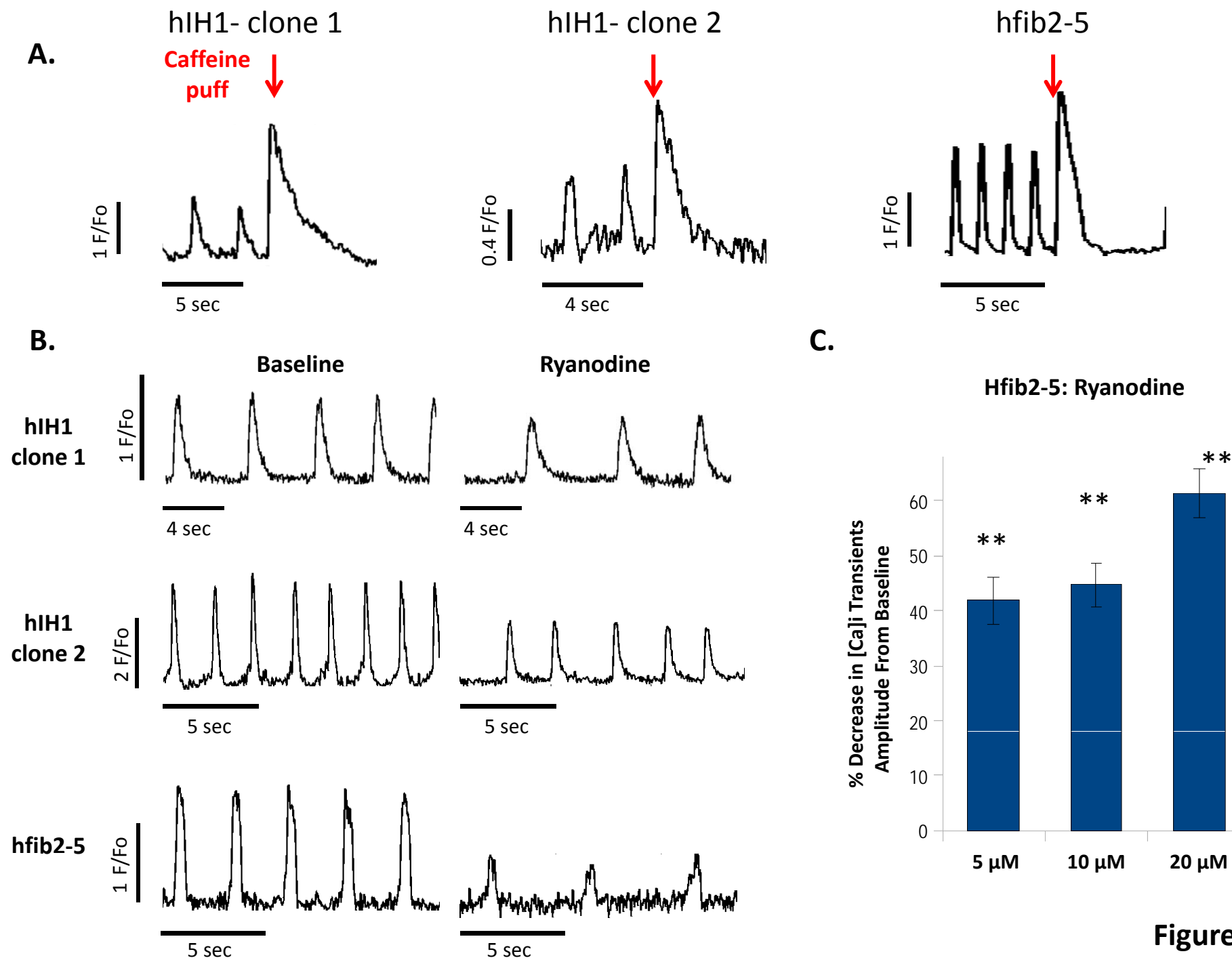

**Figure S2**

Supplement: Figure S2 — Caffeine and Ryanodine-sensitive Ca2+ stores as displayed in cardiomyocytes derived from different hiPSCs clones and lines. (A) A line-scan presenting the effect of 20 mM caffeine puff application (noted by the arrows) in hIH1 clone 1 (left), hIH1 clone 2 (middle) and hfib2-5 (right). (B) Line-scan tracings of whole-cell [Ca2+]i transients recorded from hIH1 clone 1 (top), hIH1 clone 2 (middle) and hfib2-5 (bottom) under baseline conditions (left) and in the presence of 10 µM Ryanodine (right). (C) Dose-response curve for ryanodine (5, 10, and 20 µM) displayed as the percent decrease in [Ca2+]i transients amplitude from baseline values (n = 5, **p<0.01 when mean absolute values were compared to baseline values). Abbreviations: F/Fo, fluorescence (F) normalized to baseline fluorescence (Fo); sec, seconds. (PDF) [file pone.0018037.s002.pdf]

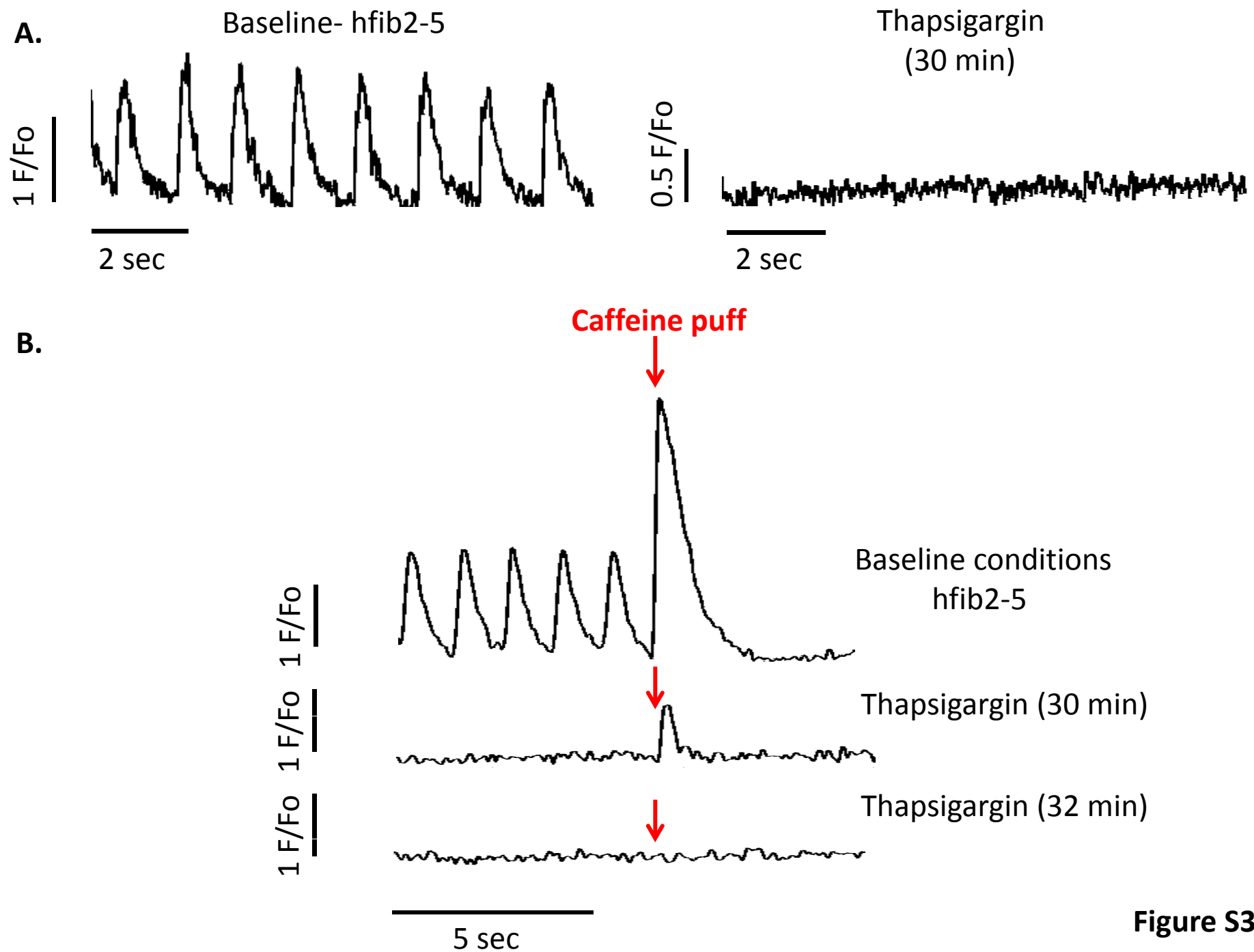

**Figure S3**

Supplement: Figure S3 — The effects of SERCA inhibition in cardiomyocytes derived from the hfib2-5 line. (A) Line-scan tracings of whole-cell [Ca2+]i transients in a representative hfib2-5 hiPSC-CM under baseline conditions (left) and after 30 minutes (right) of constant 10 µM thapsigargin exposure. (B) Caffeine-induced Ca2+ transients (denoted by arrows) under baseline conditions (upper), after 30 minutes (middle), and 32 minutes (lower) of thapsigargin exposure. Abbreviations: F/Fo, fluorescence (F) normalized to baseline fluorescence (Fo); sec, seconds. (PDF) [file pone.0018037.s003.pdf]

**A.**

Hfib2-5  
Baseline

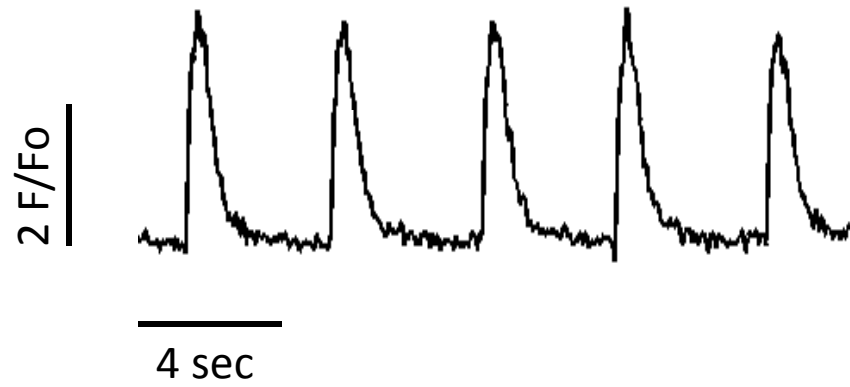

2-APB

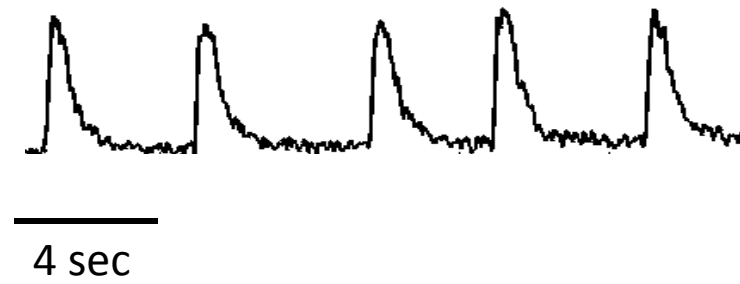

**B.**

Hfib2-5  
Baseline

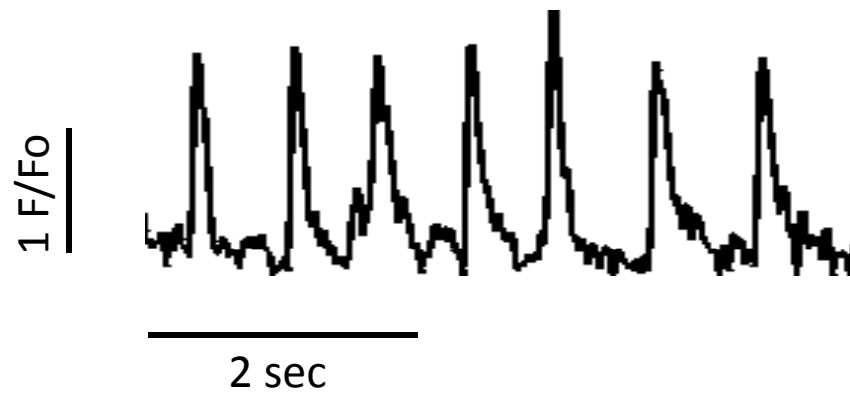

U73122

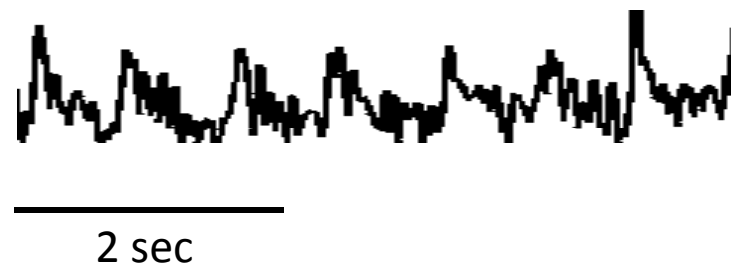

**Figure S4**

Supplement: Figure S4 — The effect of IP3R inhibition in cardiomyocytes derived from the hfib2-5 line. Whole-cell [Ca2+]i transients in representative hfib2-5 hiPSC-CMs before (left) and after (right) 2-APB (2 µM) application (A) and before (left) and after (right) U73122 (2 µM) application (B). Abbreviations: F/Fo, fluorescence (F) normalized to baseline fluorescence (Fo); IP3R, inositol-1,4,5-trisphosphate receptor; sec, seconds. (PDF) [file pone.0018037.s004.pdf]
